# QuantiFERON-TB gold test versus tuberculin skin test

**DOI:** 10.4103/1817-1737.62479

**Published:** 2010

**Authors:** Viroj Wiwanitkit

**Affiliations:** *Wiwanitkit House, Bangkhae, Bangkok 10160, Thailand. E-mail: wviroj@yahoo.com*

Sir,

I read the recent publication on the diagnosis of tuberculosis with a great interest.[1] Mardani *et al.* concluded that ‘QFT-G has been shown to be a more accurate tool to detect LTBI,’ and ‘Detecting LTBI in HIV-positive individuals showed moderate agreement between QFT-G and LTBI in our study’.[1] Indeed, there are many recent publications on this topic. Saracino *et al.* performed a similar study in Italy and reported that QFT-G might increase the identification of LTBI cases.[2] I agree that the results from this report might support an acceptable diagnostic property of QFT-G; however, there are still some concerns on this report. First, the author uses only a few subjects for assessment of the new diagnostic test. This number is questionable for statistical acceptability. Second, an important part of evaluation, the study on sensitivity and specificity was not done. Third, it is interesting to compare cost effective of the new approach to the classical tuberculin skin test. Recently, Ito *et al.* reported that ‘Estimated cost of annual QFT tests among general female nurses in Japan to prevent tuberculosis disease is very high, and annual QFT tests could not be recommended’.[3]

## References

[1] Mardani M, Tabarsi P, Mohammadtaheri Z, Chitsaz E, Farokhzad B, Hadavand F (2010). Performance of QuantiFERON-TB Gold test compared to tuberculin skin test in detecting latent tuberculosis infection in HIV- positive individuals in Iran. Ann Thorac Med.

[2] Saracino A, Scotto G, Fornabaio C, Martinelli D, Faleo G, Cibelli D (2009). QuantiFERON-TB Gold In-Tube test (QFT-GIT) for the screening of latent tuberculosis in recent immigrants to Italy. New Microbiol.

[3] Ito K (2009). Efficiency of periodic QuantiFERON-TB Gold test in hospital nurses. Kekkaku.

